# Potential of gamma/delta T cells for solid tumor immunotherapy

**DOI:** 10.3389/fimmu.2024.1466266

**Published:** 2024-08-26

**Authors:** Dantong Zhu, Xijing Ren, Wanting Xie, Jianjun Chen, Shiying Liang, Mingzhe Jiang, Junyi Wang, Zhendong Zheng

**Affiliations:** ^1^ Oncology Department, General Hospital of Northern Theater Command, Shenyang, Liaoning, China; ^2^ School of Life Sciences and Biopharmaceuticals, Shenyang Pharmaceutical University, Shenyang, Liaoning, China; ^3^ Nursing Department, General Hospital of Northern Theater Command, Shenyang, Liaoning, China

**Keywords:** gamma delta T, immunotherapy, solid tumor, adoptive cell therapy, car-t

## Abstract

Gamma/delta T (γδ T)cells possess a unique mechanism for killing tumors, making them highly promising and distinguished among various cell therapies for tumor treatment. This review focuses on the major histocompatibility complex (MHC)-independent recognition of antigens and the interaction between γδ T cells and solid tumor cells. A comprehensive review is provided regarding the classification of human gamma-delta T cell subtypes, the characteristics and mechanisms underlying their functions, as well as their r545egulatory effects on tumor cells. The involvement of γδ T cells in tumorigenesis and migration was also investigated, encompassing potential therapeutic targets such as apoptosis-related molecules, the TNF receptor superfamily member 6(FAS)/FAS Ligand (FASL) pathways, butyrophilin 3A-butyrophilin 2A1 (BTN3A-BTN2A1) complexes, and interactions with CD4, CD8, and natural killer (NK) cells. Additionally, immune checkpoint inhibitors such as programmed cell death protein 1/Programmed cell death 1 ligand 1 (PD-1/PD-L1) have the potential to augment the cytotoxicity of γδ T cells. Moreover, a review on gamma-delta T cell therapy products and their corresponding clinical trials reveals that chimeric antigen receptor (CAR) gamma-delta T therapy holds promise as an approach with encouraging preclinical outcomes. However, practical issues pertaining to manufacturing and clinical aspects need resolution, and further research is required to investigate the long-term clinical side effects of CAR T cells. In conclusion, more comprehensive studies are necessary to establish standardized treatment protocols aimed at enhancing the quality of life and survival rates among tumor patients utilizing γδ T cell immunotherapy.

## 1 Introduction

γδ T cells are a crucial cell population within the immune system, participating in both innate and non-specific immune responses. They have the ability to recognize antigens independently of MHC molecules, and are able to directly recognize a variety of molecules, such as MHC-like molecules, heat shock proteins, DNA repair related proteins, and lipid antigens. These antigens bind to receptors on γδ T cells and trigger tissue-specific activation regulated by natural killer receptors (NKR) and toll-like receptors (TLR) signaling pathways. Among these cells, the Vγ9Vδ2 T subtype is commonly employed for tumor eradication through the release of lysed particles from target cells and secretion of cytokines. Additionally, this subtype plays a significant role in immunotherapy by directly eliminating target cells while indirectly modulating other immune cell functions. γδ T cells contribute to anti-tumor effects via diverse mechanisms involving the adenosine 5’-monophosphate (AMP)-activated protein kinase (AMPK) metabolic pathway. However, under certain circumstances, γδ T cells may exhibit tumor-promoting effects primarily mediated by interleukin 17 (IL-17) pathways. Interactions among cellular networks within the tumor microenvironment also modulate the functionality of γδ T cells, including regulatory T (Treg) cells that exert inhibitory effects on γδ T cell activity. Current strategies for tumor immunotherapy involving γδ T cells encompass adoptive cell therapy and *in vitro* expansion of Vγ9Vδ2 T cell populations. In the field of cancer therapy, γδ T cells demonstrate significant potential; however, their precise role is contingent upon the distinct characteristics exhibited by different subtypes within the tumor microenvironment.

## 2 Subtype classification and function of γδ T cells

T cells are classified into αβ T cells and γδ T cells according to the differences in the types of their cell receptors. The differentiation of γδ T cells occurs subsequent to robust stimulation by T cell receptor (TCR) signals and rearrangement of the γ, δ, β chain, following their transition from CD4/CD8 double negative (DNT) cells (1). In contrast to αβ-T cells, γδ T cells exhibit distinctive attributes beyond the recognition of peptide-MHC complexes (2, 3).

Due to differences in receptor structure, there are four γδ T subtypes, Vδ1 T, Vδ2 T, Vδ3 T, Vδ5 T, different subtypes exist in different tissues and organs, resulting in different functional effects. Vδ1 T cells are predominantly found in the thymus and mucosal epithelium, constituting approximately 10% -15% of γδ T cells. In addition to recognizing CD1c and the lipid-presenting MHC-like molecule CD1d via the TCR, Vδ1 T cells can also respond to stress-induced MHC cass I associated molecules A/B (MICA/B) through the synergistic effect of TCR and natural killer cell group 2D (NKG2D). The Vδ3 T subgroup is present in liver tissue and constitutes only 0.2% of the total T cells in the human body, paired with Vγ2 or Vγ3 (4), respond to CD1d and express the degranulation marker CD107a (5). The Vδ5 T cell subpopulation predominantly resides in the peripheral blood and is stimulated by endothelial protein C receptor (EPCR) (6). Furthermore, a diverse repertoire of paired Vγ genes exists to effectively recognize ligands and elicit their functional outcomes (7).

As the predominant subtype, Vδ2 T cells are highly concentrated in peripheral blood and constitute approximately 50% to 90% of γδ T cells. They possess the ability to directly recognize and eliminate multiple tumor targets in a phosphate antigen (pAg)-dependent manner (8). Vγ9Vδ2+ T cells in fetal blood originate from the fetal thymus, whereas T cells in adult blood predominantly arise through independent production postnatally (9). Several studies have indicated that NKG2A is expressed at high levels in postnatal thymocytes, and during the early postnatal period, human Vδ2 T cells exhibit enhanced sensitivity and cytotoxicity (10). However, recent studies have demonstrated that functional Vγ9Vδ2 T cells predominantly arise in the thymus postnatally, undergo a triphasic developmental process, and mature to acquire enhanced cytokine secretion and cytotoxicity (11).

Vγ9Vδ2 T cells are frequently employed subtypes in cellular immunotherapy, exhibiting the ability to release perforin, granzyme, and other cytolytic factors while also secreting interferon-gamma (IFN-γ) and tumor necrosis factor α (TNF-α). Moreover, they indirectly modulate the activity of NK cells, B cells, CD4+, CD8+, and other immune cell populations to effectively eliminate tumors (12–15). The activation of the apoptosis pathway in tumor cells can be facilitated by the upregulation of death receptor ligands, such as FASL and tumor necrosis factor-related apoptosis-inducing ligand (TRAIL), thereby exerting potent anti-tumor effects (16). Furthermore, activation of AMPK under metabolic stress can lead to upregulated expression of butyrate 2A1 and 3A1, while Vγ9Vδ2 T cells exhibit cytotoxicity against target tumor cells by recognizing the phosphorylated antigen-induced BTN2A1-BTN3A1 complex (17–19). Vγ9Vδ2 T cells also employ Antibody-dependent Cellular Cytotoxicity (ADCC) as an effector mechanism for tumor eradication. CD16 is predominantly expressed on circulating gamma-delta T lymphocytes, which recognize target cells through the ADCC pathway upon binding of antibodies (20). Upon activation, Vγ9Vδ2 T cells upregulate CD16 expression, thereby facilitating ADCC-mediated killing of target cells following antibody-based treatments such as monoclonal antibodies targeting human epidermal growth factor receptor 2 (HER2), B-lymphocyte antigen CD20-specific monoclonal antibodies, or bispecific antibodies that bind to both TCR complexes and HER2 (21–23). Recent research has elucidated the structures of two human γδ TCR-CD3 complexes and revealed their different assembly mechanisms. The Vγ5Vδ1 TCR-CD3 complex forms a dimeric structure that is critical for T cell activation. In contrast, the Vγ9Vδ2 TCR-CD3 complex exists as a monomer with a flexible conformation, and the length of the bound peptide can modulate ligand binding and subsequent T cell activation (24). Furthermore, analogous cholesterol molecules in the transmembrane region were found to inhibit TCR signaling, which may account for the unique and irreplaceable nature of Vγ9Vδ2 T cells. These findings provide a compelling new rationale for future immune-intervention therapies.

## 3 The mechanism of action of γδ T cells

The γδ T cells not only play a pivotal role in the innate immune response, but also constitute an integral component of the non-specific immune defense. γδ T cells possess an MHC-independent antigen recognition mechanism, enabling direct recognition of antigens. They exhibit not only the ability to recognize intact polypeptide molecules but also demonstrate specific affinity towards MHC-like molecules and heat shock proteins (25).

γδ T cells demonstrate a degree of tissue-specificity in antigen recognition. γδ T cells derived from the same tissue may express analogous TCRs to recognize antigens with analogous properties, whereas γδ T cells from disparate tissues typically express disparate TCRs to identify antigens with distinct characteristics. Nevertheless, the precise mechanism by which γδ T cells recognize antigens is rather intricate and not solely dependent on the differences in TCRs, but also influenced by multiple other factors collectively (26). γδ T cells have the capacity to recognize a diverse array of antigens, including but not limited to MHC and MHC-like molecules, heat shock proteins (HSPs), phosphorylated antigens, as well as lipid antigens bound to members of the CD1 family of MHC class I-related proteins such as CD1a, CD1c, and CD1d. The interaction between these antigens and T cell receptors or NK cell receptors on the surface of γδ T cells triggers the activation of γδ T cells. Furthermore, it is possible that receptors such as NKR and TLR may also deliver co-stimulatory signals and participate in the process of activation (27). γδ T cells can be either specific or non-specific in their immune response.

The cytotoxic effect of γδ T cells on tumor cells is mediated by two primary mechanisms: direct cytotoxicity and indirect cytotoxicity. A comprehensive understanding of the interplay between these two mechanisms is crucial to unlocking the full potential of γδ T cells in cancer immunotherapy.

Direct killing is a process whereby γδ T cells recognize and bind to specific antigens on the surface of tumor cells via their surface receptors. This leads to the expression of cytotoxic molecules, such as granzyme and perforin, by γδ T cells, which subsequently release cytotoxic substances that induce apoptosis or necrosis in tumor cells (28). For example, in certain types of tumors, γδ T cells have the capacity to recognize and adhere to specific markers on the surface of tumor cells, such as MICA and MICB, which results in the release of granzyme and perforin (29).

On the contrary, the indirect elimination of γδ T cells involves their interaction with other immune cells or molecules, including the secretion of cytokines such as TNF-α and IFN-γ, to modulate the tumor microenvironment. These cytokines can impede tumor cell proliferation, facilitate immune cell activation, and augment the cytotoxicity of other immune cells against tumor cells (30, 31). Furthermore, in the context of acute myeloid leukemia, γδ T cells are capable of boosting the activity of other immune cells such as αβ T cells through the inhibition of regulatory T cells (32).

Nevertheless, γδ T cells have been demonstrated to exert protective effects in certain malignant tumors. It has been observed that IL-17+γδ T cells infiltrate tumor tissues (33). IL-17+γδ T cells expressing IL-1 β, IL-23, and/or IL-7 downregulate the expression of cc chemokine receptor 6 (CCR6), thereby promoting CCR2-dependent IL-17 production and migration of γδ T cells due to co-expression of CCR2 in these cells (34). The upregulation of IL-17 may stimulate VEGF-dependent angiogenesis (35), induce neutrophil recruitment, and employ other mechanisms to promote tumorigenesis, mobilization of pathological myeloid-derived suppressor cells(PMN-MDSCs) (36) and directly activates tumor cells through the PI3K/AKT signaling pathway (37). Subsequent research has demonstrated that tumor-infiltrating Vδ1+ cells secrete IL-17, which is associated with higher rates of recurrence, lymph node metastasis and mortality (38). Conversely, in breast cancer, tissue-resident Vδ1+ T cells have been demonstrated to favor cytolysis and IFN-γ production over IL-17 secretion (39). Furthermore, glioblastoma(GBM) patients with high levels of IL-17 expression exhibited longer survival compared to those with low levels of IL-17 expression (40). These findings indicate that the cytokine profile of the γδ T cell subpopulation is significantly influenced by the tumor microenvironment and cellular interactions.

**Figure 1:**
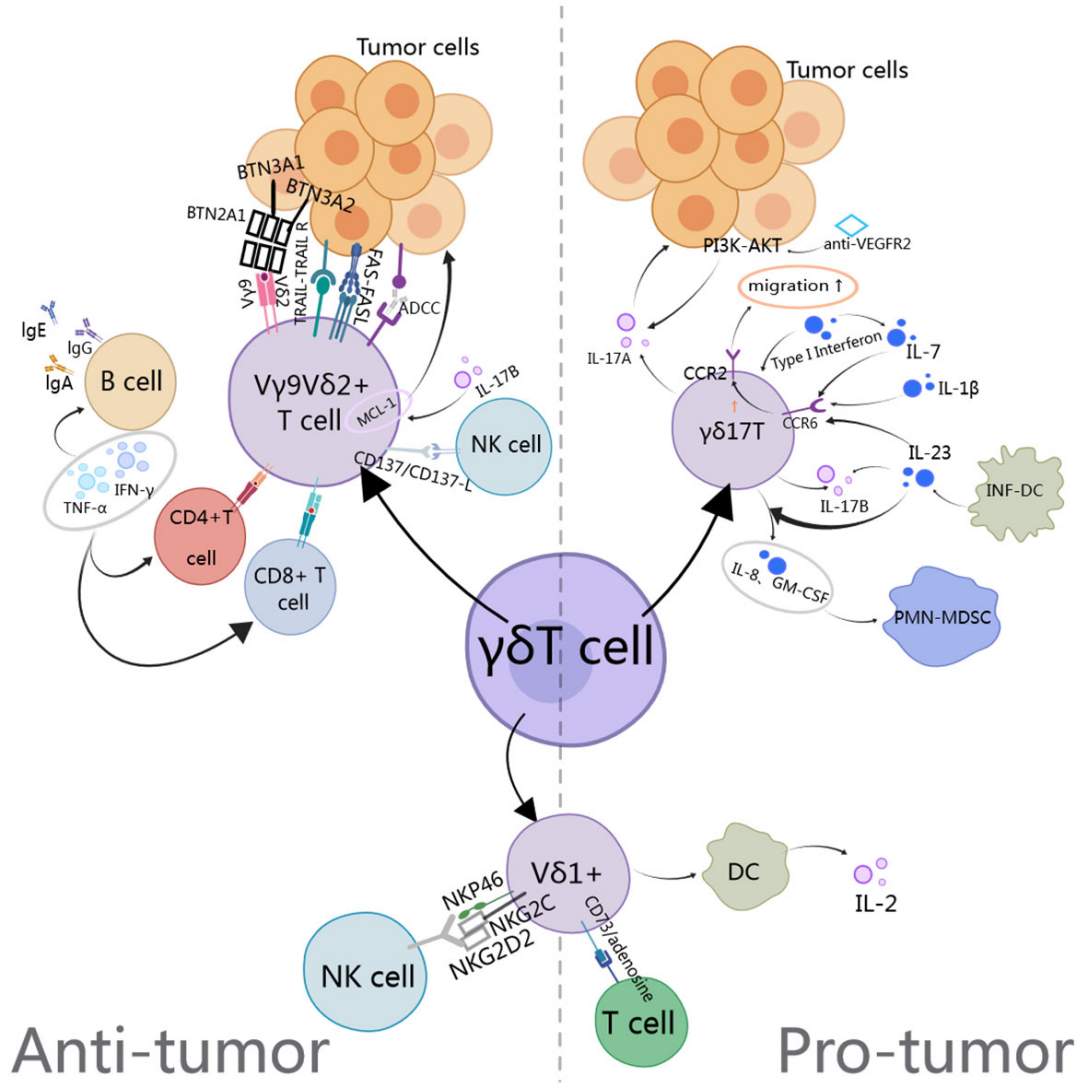
The anti-tumor and pro-tumor mechanisms of gamma delta T cells involve the TCR PI3K, AKT, INF-DC (Inflammatory dendritic cells), PMN-MDSC, CCR2 (Chemokine receptor-2), and BTN.

One of the current methods of using γδ T cells for tumor immunotherapy is adoptive cell therapy, which involves *in vitro* expansion of Vγ9Vδ2 T cells through adoptive transfer, synthesis of phosphate antigen analogues, or *in vivo* stimulation with amino succinic acid (41). The *in vitro* expansion of Vγ9Vδ2 T cells is easy for Vδ1 cells. Amino bisphosphonates such as pamidronate and zoledronate, as well as synthesized phosphate antigen analogues, serve as ligands for γδ TCR and can induce the production of pyrophosphate intermediates in tumor cells by upregulating the mevalonate pathway (42). Both domestically and internationally γδ T autologous/allogeneic reinfusion has been used in preclinical and clinical studies on multiple cancer types, including pancreatic cancer (43), cholangiocarcinoma (44), non-small cell lung cancer, gastric cancer, etc. (**Table 1**). The research results indicate that *in vitro* expanded Vγ9vδ2 T cells have excellent tumor killing ability, and adoptive therapy is safe and well tolerated, but its clinical efficacy still needs to be verified. For this significant difference, researchers believe that the pharmacokinetics of bisphosphonates may hinder their systemic efficacy in tumor immunotherapy (45). The human body rapidly removes bisphosphonates and other drugs from circulation through renal excretion and bone absorption, making it difficult to retain them in tumor tissue (46). Consequently, the extent of γδ T cell activation by bisphosphonates in tumor infiltration remains elusive. Similarly, limited evidence exists to support the transportation or retention of *in vitro* activated γδ T cells within tumors (47). Indeed, the activation of Vδ2^+^ T cells by pAg is closely associated with their depletion/energy status, and due to the antigen-presenting capability of Vδ2+ T cells, it can induce the upregulation of chemokine receptors involved in lymph node homing (15, 48–51). In view of the dual function of γδ T cells in tumor regulation, we have provided a comprehensive summary of our observations, as illustrated in **Figure 1**.

**Table 1:** Summary of Clinical Trials on Gamma Delta T Cell Therapy for Tumors.

Innate T cell type	Start Year	Targeting cancer	Outcome	Main ID	Other procedure	Publications
Autologous gamma/delta T lymphocytes	2006	Non-Small Cell Lung Cancer	6SD,6PD	JPRN- C000000336		(108, 128)
γδ T cells	2007	Bone metastases		JPRN- UMIN000000628	Radiotherapy	
Gamma/delta T cell	2007	Colorectal cancer		JPRN- UMIN000000854		(129)
Gamma/delta T cell	2007	Hepatocellular Carcinoma		NCT00562666		
Autologous gamma/delta T cell	2007	Pancreatic cancer		JPRN- UMIN000000931	Resection and gemcitabine	
Autologous gamma/delta T cell	2008	Esophageal cancer		JPRN- UMIN000001419		
Autologous gamma/delta T cell	2008	Hepatocellular carcinoma		JPRN- UMIN000001418		
Autologous gamma/delta T cell	2008	Intrahepatic cholangiocarcinoma or biliary tract cancer		JPRN- UMIN000001417		
Autologous gamma/delta T cell	2009	Stage2A (T2N0,T3N0) esophageal cancer		JPRN- UMIN000002839	After resection	
Autologous gamma/delta T cell	2010	CD20-positive B-cell lymphoma		JPRN- UMIN000003641	Rituximab	
Autologous gamma/delta T cell	2010	Refractory gastric cancer with ascites		JPRN- UMIN000004130		(28)
Autologous gamma/delta T cell	2010	Hepatitis C virus-related hepatocellular carcinoma		JPRN- UMIN000004583	Radiofrequency ablation therapy	
2-Methyl-3-Butenyl-1- Pyrophosphate- Stimulated Gamma Delta	2011	Stage IV Renal Cell Carcinoma		JPRN- UMIN000004482		
Zoledronate-expanded autologous gamma/delta T cells	2011	Non-small cell lung cancer refractory to standard treatment.		JPRN- UMIN000006128		
2-Methyl-3-Butenyl-1- Pyrophosphate- Stimulated Gamma Delta	2011	PSA biochemical failure after radical prostatectomy		JPRN- UMIN000006617		
Auto-gamma/delta T cell	2012	Multiple myeloma		JPRN- UMIN000007878		
Gamma-delta T Cells	2012	Epithelial Ovarian Cancer IClyCO		NCT01606358		
Autologous gamma/delta T cell	2012	Esophageal cancer		JPRN- UMIN000008097	Docetaxel/cisplatin/fluorouracil (DCF)	
Gamma-delta T cell	2012	Malignant tumor		JPRN- UMIN000009422	Immunotherapy	
2-methyl-3-butenyl-1- pyrophosphate-stimulated gamma delta T cells	2013	Non-invasive bladder cancer		JPRN- UMIN000010942		
Autologous gamma/delta T cell	2013	Hepatocellular Carcinoma		JPRN- UMIN000011184		
Gamma Delta T Immune- Cells	2014	Excision Impossible Pancreatic Cancer		JPRN- UMIN000013794		
PepTivator(R) WT1- added autologous gamma/delta T cell 2-Methyl-3-Butenyl-1-	2014	Cancer		JPRN- UMIN000015410		
Pyrophosphate- Stimulated Gamma Delta T Cells	2015	Advanced Renal Cell Carcinoma		JPRN- UMIN000016793		
γδ T + DC-CIK	2015	Breast cancer		NCT02418481		
γδ T cells	2015	Hepatocellular liver cancer		NCT02425735	tumor reducing surgery	
γδ T + DC-CIK	2015	Non Small Lung Cancer		NCT02425748		
γδ T+CIK	2015	Gastric cancer		NCT02585908		
Anti-CD19-CAR γδ T	2016	B-Cell Lymphoma, ALL and CLL		NCT02656147		
γδ T cells	2017	Locally Advanced Pancreatic Cancer		NCT03180437	IRE surgery	
γδ T cells	2017	Breast Cancer		NCT03183206	Cryosurgery, IRE surgery,surgery	
γδ T cells	2017	Liver Cancer		NCT03183219	Cryosurgery or IRE surgery	
γδ T cells	2017	Lung Cancer		NCT03183232	Cryosurgery or IRE surgery	
Expanded/Activated Gamma Delta T-cell	2017	ALL/AML/CML		NCT03533816	Hematopoietic Stem Cell Transplantation and Cyclophosphamide	
Autologous gamma/delta T lymphocytes	2019	Advanced Hepatitis B Related Hepatocellular Carcinoma		NCT04032392		
Adoptive Cell Transfer of NKG2DL-targetting Chimeric Antigen Receptor-grafted Gamma Delta T cell	2019	Nasopharyngeal Carcinoma/Colorectal Cancer/Sarcoma/Triple Negative/Breast Cancer/Prostate Cancer/Gastric Cancer		NCT04107142		
Gamma-Delta T Cell	2019	Glioblastoma DRI		NCT04165941		
Gamma delta T cells	2020	lung cancer		ChiCTR20000291 02		
Ex-vivo expanded allogeneic γδ T cells	2020	Phase 1 Hepatocellular Carcinoma		NCT04518774		
Ex-vivo expanded allogeneic γδ T cells	2021	Non-Hodgkin’s Lymphoma (NHL)/Peripheral T Cell Lymphomas (PTCL)		NCT04696705		
CAR - γδ T	2021	CD7 Positive T cell-derived malignant tumors		NCT04702841		
ADI-001 Anti-CD20 CAR- engineered Allogeneic Gamma Delta T Cells	2021	Adults With B Cell Malignancies		NCT04735471	Cyclophosphamide/Fludarabine	
LAVA-1207(Humanised					
bispecific immunoglobulin					
VHH fragments against PSMA and Vgamma9Vdelta2 T-cell receptor)	2021	Therapy refractory metastatic castration resistant prostate cancer		EUCTR2021-001789-39-NL		
Gamma Delta T-cell	2021	Acute Myeloid Leukemia at High Risk of Relapse		NCT05015426		
CAR–γδ T cells	2022	AML		NCT05388305		
Allogeneic Expanded Gamma Delta T Cells	2022	Relapsed or Refractory Neuroblastoma Aflac-NBL-2002		NCT05400603	GD2 Chemoimmunotherapy	
ACE1831(Allogeneic CD20-conjugated Gamma Delta T-cell)	2022	Relapsed/Refractory CD20-expressing B-cell Malignancies		NCT05653271		
Allogeneic or Autologous γδ T Cells	2022	Recurrent or Newly Diagnosed Glioblastoma		NCT05664243		
Gamma Delta T Cell	2023	Stage 4 Metastatic Non-Small Cell Lung Cancer		NCT06069570	Low Dose Radiotherapy	
Allogeneic CAR Gamma- Delta T Cells	2023	Relapsed/Refractory Solid Tumors		NCT06150885		(111)
Autologous Gamma Delta T Cells Genetically Engineered With a	2023	Metastatic Castration Resistant Prostate Cancer		NCT06193486		
Chimeric Receptor Autologous γδ T adoptive immune cells	2023	Inoperable or metastatic biliary pancreatic malignancies		ChiCTR23000747 94	Gemcitabine-based standard first-line chemotherapy	
Allogeneic Gamma-delta T Cells	2024	Hepatocellular Carcinoma Resistant to PD-1 Monoclonal Antibody		NCT06364800	Targeted Therapy and Immunotherapy	
Allogeneic Gamma-delta T Cells	2024	Hepatocellular Carcinoma		NCT06364787	Targeted Therapy and Immunotherapy	
Anti-EGFR Conjugated Gamma-delta T Cell	2024	Metastatic Solid Tumor/Locally Advanced Solid Tumor		NCT06415487		

## 4 Application and mechanism of different kinds of CAR-γδ T cells

At the time of writing this article, in the context of solid tumor γδ T immunotherapy, aside from autologous cell adoptive transfer therapy, Adicet Bio, BMS (Bristol Myers Squibb), and Century Therapeutics have developed clinically effective application CAR-γδ T treatment methods. Furthermore, Gadeta, IN8bio, and CytoMed Therapeutics in Singapore have all conducted clinical trials related to engineering γδ T cells.

CAR-mediated γδ T cells demonstrate heightened efficacy against tumor cells expressing engineered receptor structural target antigens, encompassing Vδ1 and Vδ2 subsets. It is noteworthy that CAR-γδ T cells also retain their antigen presentation function *in vitro* (52). Concurrently, disialoganglioside (GD2)-directed CAR-γδ T cells also exhibit heightened cytotoxic potential compared to unmodified γδ T cells (53).

CAR-Vγ9Vδ2 T cells targeting mucin-1 have also been engineered to suppress the subcutaneous growth of mouse metastatic gastric cancer cell lines. In comparison to αβT cells, CAR-Vγ9Vδ2 T cells exhibit enhanced *in vitro* cytotoxicity. However, prolonged exposure to tumor cells appears to necessitate IL-2 supplementation for the restoration of their cytotoxic activity (54). Furthermore, the utilization of chimeric antigen receptors (CARs) targeting cell surface NKG2D ligands in Vγ9Vδ2 T cells has demonstrated an extended survival period in murine xenograft models of ovarian cancer (55). Clinical trials have also been conducted to assess the clinical effectiveness of allogeneic γδ T cell immunotherapy targeting NKG2D ligands in patients with recurrent and/or refractory metastatic solid tumors.

Vδ1 T cells exhibit a diminished propensity for activation-induced cell death, and, in comparison to Vδ2 T cells, demonstrate prolonged *in vivo* persistence (56). The Phase I trial results of CD20-targeted CAR Vδ1 T cells in humans are promising (57), while another CAR Vδ1 T cell product targeting glycan-3 (overexpressed in various solid tumor types) has been developed and further modified to produce soluble IL-15 (58, 59).

In addition, considering that different subtypes of γδ T cells may be regulated differently in TME, and concurrent chemotherapy or radiotherapy with cell therapy may affect the sensitivity of tumor cells to γδ T cells (13). Studies have shown that anti CTLA4 antibody therapy increases the frequency of Vδ2+ T cells in melanoma patients (60), suggesting that different drug combinations may have a positive effect on the anti-tumor function of γδ T. Of course, there are also negative cases (61), so activation is necessary γδ T cell first may be more important (62). Some companies also hope to regulate endogenous γδ T cells levels in patients by using monoclonal antibodies (MABs) and bispecific antibody drugs. Adaptate and ImCheck Therapeutics activate Vγ9Vδ2 T through monoclonal antibody agonists cells, while Lava activate Vγ9vδ2 T through a specific membrane antigen targeting the prostate gland as a cell conjugate (63).

**Figure 2:**
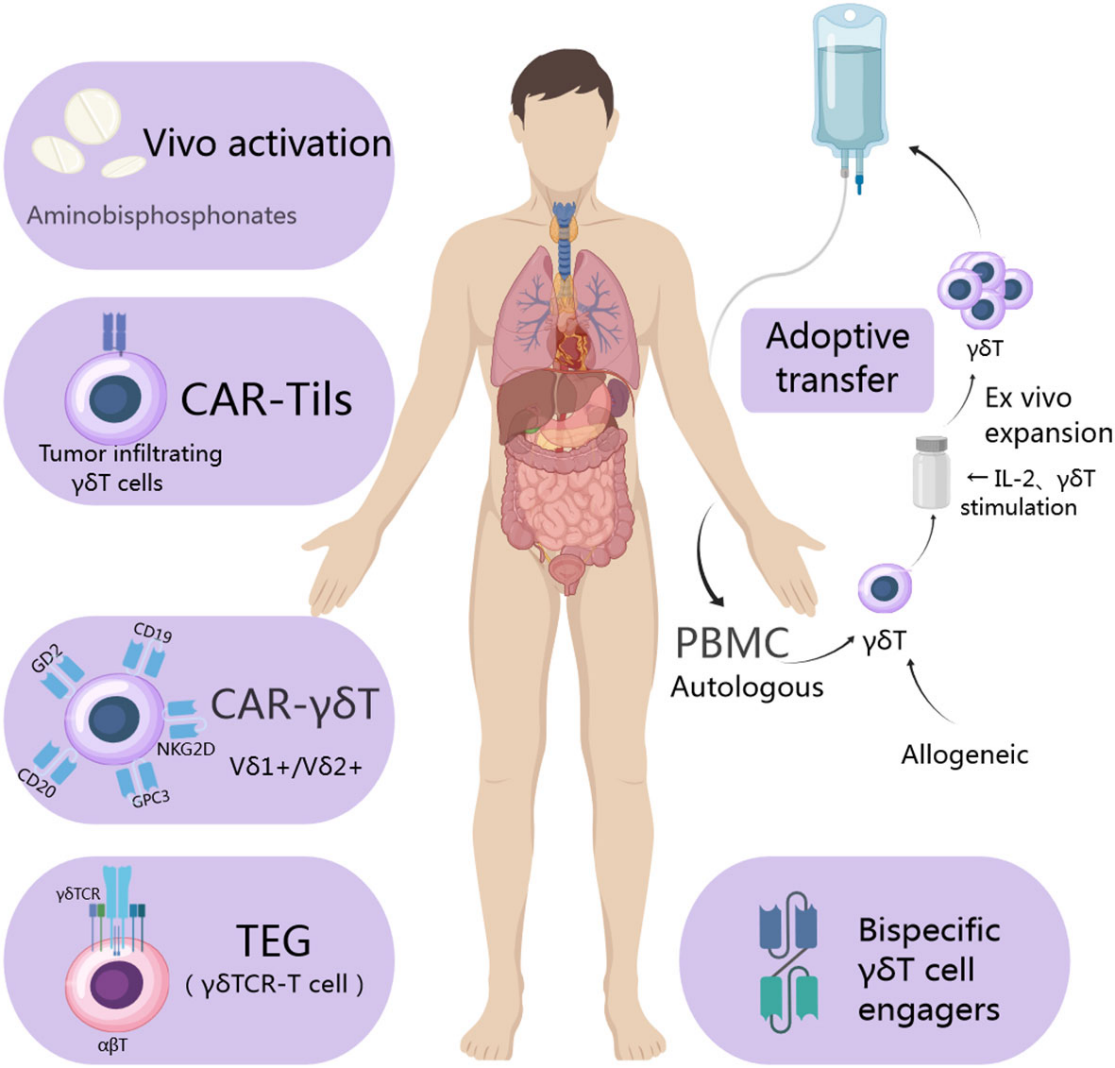
Utilization of γδ T cells in the treatment of tumor cells involves isolating allogeneic γδ T cells from a healthy donor and autologous ones from the patient’s own cells. Following isolation, various strategies such as CAR utilization, T cell receptor (TCR) transfer, and cell engager are employed to expand and engineer γδ T cells. Subsequently, these engineered γδ T cells are administered directly to the patient or utilized as a form of immunotherapy to achieve their therapeutic function.

By combining the extracellular domain of tumor-responsive Vγ9Vδ2 TCR with CD3 binding fragments (GABs), bispecific molecules can be engineered to mimic the mode effect mediated by Vγ9Vδ2 TCR. In a subcutaneous bone marrow tumor xenograft model, GABs significantly inhibit *in vivo* tumor growth. Furthermore, T cell adapter bispecific antibodies may enhance the effectiveness of adoptive transfer of γδ T cells. The newly developed Vδ2 x PD-L1 can redirect Vγ9Vδ2 T cells to PD-L1 positive tumor cells and induce their killing. The binding of Vδ2 x PD-L1 with adoptive metastatic Vγ9Vδ2 T cells inhibits the growth of existing tumor xenografts while increasing the number of Vγ9Vδ2 T cells on the tumor bed (64). Lava-051 is a 27kD humanized bispecific single domain antibody (VHH) that directly targets CD1d, and the Vγ9Vδ2 TCR chain mediates efficient killing of CD1d-expressing tumor cells. Furthermore, they also found the stimulation of Vγ9Vδ2 T cells through cross-linking with prostate-specific membrane antigen (PSMA) induces potent and selective killing of PSMA-positive tumor cells. In brief, γδ T cells’ current applications are illustrated in **Figure 2**.

Hence, to advance novel immunotherapy approaches and expand treatment options for cancer patients, further investigation into the role of γδ T lymphocytes in solid tumor immunotherapy is of paramount importance.

## 5 Effectiveness of gamma/delta T-cell therapy in solid cancers

γδ T cells not only exert anti-tumor effects by recruiting T cells and natural killer cells, secreting cytokines, but also promote tumor progression and spread due to the infiltration of the tumor microenvironment and the utilization of cytotoxic mechanisms by cancer cells. Here we summarize the effectiveness of γδ T cells in several types of solid tumors.

### 5.1 Breast cancer

The incidence of female breast cancer has surpassed that of lung cancer, making it the most prevalent form of cancer worldwide (65). With the ongoing urbanization in China, there is a potential for an annual increase in the incidence and mortality rates of young women (66).

The progression of breast cancer is influenced by the production of γδ IL-17A, which is a key factor for various subtypes of γδ T cells (67). In breast tumor-bearing mice, type I IFN directly suppresses the activity of tumor-infiltrating γδ17 T cells, while indirect inhibition of γδ17 T cell-activating factor IL-7 reduces IL-17A secretion through two pathways, thereby impeding tumor progression (68). γδ T cells can also induce the upregulation of MICA/B and ICAM1 expression on the surface of sensitive strain SkBr7, facilitating its interaction with NKG2D receptors on the surface of γδ T cells, leading to alterations in intracellular protein kinases such as AKT and extracellular signal-regulated kinase(ERK) that are associated with cellular proliferation. Concurrently, the phosphorylation levels of signal transduction and transcription activating factor 3 (STAT3) decrease, while the expression levels of pro-apoptotic molecules PARP and Caspase3 increase. γδ T cells exhibit a significant inhibitory effect on tumor formation in the sensitive strain SkBr7, as evidenced by accelerated tumor cell apoptosis, inhibited angiogenesis, and reduced tumor burden. Furthermore, the secretion of chemokines and infiltration of tumor macrophages also contribute to these processes. They collectively bolster tumor immune surveillance and augment their anti-tumor efficacy (69). γδ T cells stimulate the upregulation of MHC class I and CD54/intercellular adhesion molecule-1 (ICAM-1) expression in cancer stem cell (CSC)-like cells, thereby enhancing the susceptibility of CD8+ T cells to antigen-specific killing (70, 71). Conversely, through the induction of IL-17 expression in γδ T cells, IL-1 β facilitates the lymph node and lung metastasis of cancer cells (36). Human Vδ1+ T cells suppress dendritic cell (DC) maturation, diminish IL-2 production, and contribute to immune evasion in tumors (72). A high dosage of anti-VEGFR2 can stimulate the PI3K-AKT pathway and promote the expression of IL-17A (73). Furthermore, the Vδ1+ subgroup is capable of suppressing conventional T cell proliferation and facilitating tumor progression via CD73/adenosine-dependent pathways (74).

Research on the mechanism of γδ T cell-mediated tumor killing via ADCC reveals that the combination of trastuzumab and phosphoantigen-activated γδ T lymphocytes augments the efficacy of trastuzumab against HER2+ breast carcinoma cell lines *in vivo* (22). Although the combination of neoadjuvant therapy and phosphate may not lead to an overall local tumor response, zoledronic acid demonstrates significant efficacy in breast cancer patients with low γδ T cell frequency (75, 76). Furthermore, numerous clinical trials with unpublishable results are currently in progress (NCT02418481, NCT03183206, NCT04107142).

### 5.2 Head and neck cancer

Neuroblastoma(NB) is one of the most common solid tumors in children (77), with poor prognosis and a 5-year survival rate of less than 50% in high-risk NB patients (78).

Unlike other tumors, the amplified *in vitro* Vδ1+and Vδ1-Vδ2-γδ T cells also exhibit clinically effective cytotoxicity towards them (79). Besides, Vδ2+ T cells can be manipulated to link to an endodomain that provides the NKG2D adaptor protein DAP10 co-stimulation but no TCR signal with a CAR comprising conventional ectodomain targeting GD2, which may avoid cytotoxicity to normal cells expressing same target (80). However γδ T adoptive immunotherapy requires a sufficient number of cells to achieve therapeutic effects, and IL-15 can increase the survival rate of human γδ T cells by regulating the expression of myeloid cell leukemia 1 (Mcl-1) (81). Anti GD2- γδ T can also activate downstream signaling domains by recognizing tumor PAG antigen or GD2, exerting specific anti-tumor effects and effectively cleaving NB cells without affecting normal cells (82). However, γδ T cells can also facilitate the proliferation and migration of NB tumor cells by generating IL-17 and releasing cytokines such as IL-17A (83).

### 5.3 Tumors of the digestive system

#### 5.3.1 Pancreatic cancer

Pancreatic cancer is characterized by rapid progression, resulting in a high mortality rate and a median survival time of less than one year (84, 85). Research has demonstrated that the combination of γδ T cells, IL-2, and [(HER2) 2Vγ-9] was employed in the treatment of tumor-bearing models established using the pancreatic cancer cell line PancTu-I and SCID Beige mice, resulting in significant inhibition of tumors in all mice (21). IL-17B induces the upregulation of Chemokine Ligand 20 (CCL20), chemokine ligand 1(CXCL1), IL-8, and Trefoil Factor 1 (TFF1) chemokines via the ERK1/2 pathway, thereby facilitating cancer metastasis and enhancing viability in distant organs (86). Receiving IL-17RB monoclonal antibody treatment has the potential to inhibit tumor metastasis and enhance overall survival rates (87). PDA infiltration leads to γδ T cell overexpression, resulting in ligand depletion and subsequent activation of αβ T cells, which exerts tumor-protective effects. Additionally, PDA expresses programmed cell death ligand 1 (PD-L1) and galactin-9 to inhibit cytotoxic T cells (88). In addition, endothelin can enhance the infiltration of NK, NKT, and γδ T cells while reducing PMN-MDSCs infiltration, thereby modulating the tumor immune microenvironment and suppressing tumor growth (89).

#### 5.3.2 Cholangiocarcinoma

As a primary liver tumor, cholangiocarcinoma demonstrates high malignancy and heterogeneity, resulting in a 5-year survival rate of only 20% and a poor prognosis (90).

The clinical efficacy of γδ T cells in treating cholangiocarcinoma has been demonstrated. Based on current clinical trial results, there have been reports of eight instances where infusion of allogeneic Vδ2+ T cells successfully reduced lymph node metastasis in patients with cholangiocarcinoma (44). Furthermore, the combination of local ablation and γδ T adoptive transfer has exhibited promising clinical efficacy in the treatment of intrahepatic cholangiocarcinoma(ICC) (91).

#### 5.3.3 Colorectal cancer

The incidence and mortality rates of colorectal cancer are among the highest in the world, with the highest incidence rate observed in East Asia (92, 93).

γδ T cells represent the predominant lymphocyte subset in colorectal cancer(CRC), and their interaction with NK cells via NK ligands constitutes a crucial mechanism for γδ T cells to exert anti-tumor effects. Experiments have shown that the killing effect of Vγ9Vδ2 T cells on most colon cancer cells is related to the accumulation of IPP and the expression of ICAM-1 (94). The engagement of NKG2D receptors with MICA/B and ICAM-1 on the surface of colon cancer cells triggers activation, leading to the release of perforin and granzyme B by activated γδ T cells. These cytotoxic effector molecules, along with IFN-γ and TNF-α cytokines, mediate the elimination of colon cancer cells through pathways such as TRAIL and FAS/FASL (95). While Vγ9Vδ2 T cells exert cytotoxic effects on most colon cancer cell lines, Vδ1T cells demonstrate superior *in vitro* cytotoxicity against adherent and spherical human colon cancer cells compared to Vδ2T cells (96). The proportion of tumor-infiltrating Vδ1+T cells increases, which can enhance tumor eradication by engaging NK cells via ligands such as NKP46, NKG2C, and NKG2D (97, 98). On the contrary, γδ17 T cells play a contrasting role in the context of colon cancer. Upon activation by INF-DC, these cells secrete IL-8, tumor necrosis factor, and GM-CSF, thereby promoting the accumulation of immunosuppressive PMN-MDSCs and facilitating the progression of colon cancer. Furthermore, in addition to infiltrating γδ T cells, mouse experiments show those expressing Vγ4+ and Vγ6+ also contribute to tumor progression (48, 99).

### 5.4 Respiratory system tumors

The progression of lung cancer may also be associated with IL-17. Research indicates that in early non-small cell lung cancer, the primary source of IL-17 is tumor-infiltrating γδ T cells, and a higher abundance of γδ T cells has been observed in lymph node metastases of non-small cell lung cancer patients (100). Furthermore, the transfer of γδ T cells and supplementation with IL-17A has been shown to diminish the size of murine lung tumors and decrease the number of lung tumor foci (101). Enhanced anti-tumor activity against pulmonary melanoma is attributed to the heightened presence of γδ17 T cells in aged lungs (102). The experimental results demonstrate that γδ T cell immunotherapy upregulated the release of perforin and granzyme B through the Bax/Bcl-2 signaling pathway. Additionally, γδ T cell-mediated lysis of A549 cells involves the PI3K/AKT pathway, thereby augmenting cytotoxicity against lung cancer cells (103). In addition, Vδ1 T cells isolated from NSCLC patients exhibit enrichment of CD45RA and CD27 TEM subsets, which are potent producers of IFN-γ. Conversely, the absence of IL-10 amplifies cellular proliferation, resulting in an elevated frequency of IL-17+ γδ T cells and an increased level of IL-17A in murine malignant tumors (MPEs). However, further validation is necessary in human immunology. It is evident that γδ T exhibits both anti-tumor and pro-tumor effects simultaneously (104, 105). Human Vδ1 T cells consistently display a Tc1 phenotype associated with tumor rejection during *in vitro* activation (106). Wuhan YZY Biopharmaceutical Co., Ltd. has developed a bispecific antibody (PD-L1 x CD3) Y111 that targets PD-L1 and CD3, effectively facilitating the interaction between T cells and tumor cells expressing PD-L1. Experiments have demonstrated that Y111 can enhance the cytotoxicity of Vγ9Vδ2 T cells against various non-small cell lung cancer cells and suppress the growth of xenografts in NPG mice (107).

Given the efficacy of γδ T cells in the treatment of non-small cell lung cancer by adoptive transfer therapy in humans has been limited (108). This is likely due to the predominant focus on Vδ2+ T cells in research efforts. For example, a multicenter Phase II study conducted in 2020 involved autologous infusion therapy with Vγ9Vδ2 T cells for 25 lung cancer patients, resulting in partial relief observed in 1 case (109), which means we truly need more research on how to improve the therapeutic efficacy of γδ T cells for cancer patients.

### 5.5 Other solid tumors

There are kinds of engineering γδ T cells being studied to get over various tumors. In addition to the ones mentioned above, CD-19 targeted CAR-γδ T cell therapy in B-lymphocytic tumor patients showed a complete remission rate (110), and CD20 targeted CAR-γδ T cell therapy is also promising with complete remission observed in 4 out of the initial 6 patients (57), In hepatocellular carcinoma(HCC) xenograft models, single infusion of GPC-3 specific transgenic T cells (CAR) and soluble IL-15 engineered ready-made Vδ1+ γδ T cells can control tumor growth (59) Besides, Lava Therapeutics is currently conducting a Phase I/IIa clinical trial to assess Lava-1207, a Gamma body, in patients with metastatic castration-resistant prostate cancer and the efficacy remains to be observed(NCT05369000).

## 6 Discussion

γδ T cells recognize antigens independently of MHC, and their substantial anti-tumor potential is garnering increasing attention. Currently, numerous global clinical trials have been conducted on γδ T cells (28, 111, 112), particularly demonstrating promising results for the allogeneic transfusion of anti-tumor cell products. The current understanding of the classification of human γδ T cells and their specific subtypes, as well as their interactions with solid tumor cells, remains incomplete. Furthermore, the characteristics and mechanisms of tumor cells in reversing the regulation of γδ T cell function have also been elucidated (113, 114). Currently, the investigation of the roles played by various subtypes of γδ T cells in tumor development and migration represents a prominent focus in fundamental research, through molecules associated with apoptosis such as FAS/FASL pathways, BTN3A-BTN2A1 complexes, and their interactions with CD4, CD8, and NK cells have emerged as potential targets for activating Vγ9Vδ2 T cells (42, 115). As the fetus develops into an adult, Vγ9Vδ2 T gradually becomes the main subpopulation of peripheral blood γδ T cells. Compared to Vγ9Vδ2 T cells isolated from umbilical cord blood, isolating from peripheral blood monocytes is more convenient and effective. Due to the requirements for cell quantity, survival rate, and cytotoxicity in cell adoptive therapy, it is necessary to perform *in vitro* expansion of Vγ9Vδ2 T. However, according to currently published studies, the existing expansion methods cannot fully guarantee consistent purity. Hence, the exploration of antibodies and small molecule drugs that target the interaction between Vγ9Vδ2 T cells and cancer cells represents a promising avenue for research. However, their clinical applicability remains to be assessed.

Immune checkpoint inhibitors, such as PD-1 and PD-L1 inhibitors, are currently a focal point in the field of tumor treatment. Furthermore, in the realm of γδ T cell research, it has been observed that the utilization of PD-1 inhibitors can augment their cytotoxic activity against tumor cells (116, 117), and this synergistic effect will provide more options for the treatment of tumors by γδ T cells. The current cutting-edge research on CAR-T methods has also been extended to the application of γδ T cells. CAR-γδ T cells exhibit tissue-specific residency and distinct *in vivo* homing capabilities, thereby offering broad prospects for γδ T cell therapy (118, 119). Correspondingly, the clinical trial proportion of related products is relatively high. However, practical considerations regarding the manufacturing and clinical aspects of CAR-γδ T cells have also garnered significant attention. For instance, the persistent depletion of γδ T cells in the organism may result in residual tumor progression, necessitating multiple infusions of CAR-γδ T cells to augment the host’s immunogenicity towards CAR (120), besides, the single recognition mechanism of antigens in CARs results in poor ability to distinguish between normal cells and tumor cells, exhibiting targeted non tumor cell toxicity. Especially in the treatment of T-cell malignancies, the interaction between CAR-T cells targeting common antigens and lethal T-cell aplasia make clinical application difficult (121). To date, there has been no formal study comparing the toxicity of CAR-T cells in αβ T, γδ T, and NK cells, which also impedes the widespread application of such products. Thus, further research is warranted to enhance our understanding of the role of CAR-γδ T cells in the tumor microenvironment characterized by immunosuppression, hypoxia, and metabolic competition, as well as to investigate the long-term clinical implications of CAR-T cell therapy. These efforts will strengthen the rationale for utilizing CAR-γδ T cell therapy in the treatment of solid tumors.

Due to the potential tumorigenic effect of total gamma delta T cells, they may stimulate the activation of immunosuppressive regulatory T cells, thereby promoting tumor growth (122, 123). Additionally, γδ T cells are associated with various autoimmune diseases (124–127), and further research is needed to investigate the safety of γδ T cell therapy in future studies. Our research center has rigorously studied γδ T cell infusion (ChiCTR2300074794) for safety and efficacy, with ongoing trials showing promising results. Based on current data, we are highly confident in this treatment approach.

In conclusion, further comprehensive and detailed research is needed for the immunotherapy of solid tumors with γδ T cells, along with the establishment of standardized screening and treatment protocols to enhance both the quality of life and survival prospects for tumor patients.
